# Antithrombotic Therapy and Bleeding Risk Among Young Adults After Ischemic Stroke

**DOI:** 10.1001/jamanetworkopen.2026.35826

**Published:** 2026-09-24

**Authors:** Anne Rasing, Esmée Verburgt, Merel S. Ekker, Mijntje M. I. Schellekens, Maikel H. M. Immens, Jamie I. Verhoeven, Esther M. Boot, Mayte E. van Alebeek, Paul J. A. M. Brouwers, Renate M. Arntz, Gert W. van Dijk, Inge W. M. van Uden, Rob A. R. Gons, Tom den Heijer, Julia H. van Tuijl, Karlijn F. de Laat, Anouk G. W. van Norden, Sarah E. Vermeer, Marian S. G. van Zagten, Robert J. van Oostenbrugge, Marieke J. H. Wermer, Paul J. Nederkoorn, Henk Kerkhoff, Fergus A. Rooyer, Frank G. van Rooij, Ido R. van den Wijngaard, Tim J. F. ten Cate, Anil M. Tuladhar, Edo Richard, Frank-Erik de Leeuw, Nina A. Hilkens

**Affiliations:** 1 Department of Neurology, Radboud University Medical Centre, Radboud Institute for Medical Research and Innovation, Donders Centre for Medical Neurosciences, Nijmegen, the Netherlands; 2 Department of Neurology, Rijnstate Hospital, Arnhem, the Netherlands; 3 Department of Neurology, Gelre Hospital, Apeldoorn, the Netherlands; 4 Department of Neurology, Medisch Spectrum Twente, Enschede, the Netherlands; 5 Department of Neurology, Canisius-Wilhelmina Hospital, Nijmegen, the Netherlands; 6 Department of Neurology, Catharina Hospital, Eindhoven, the Netherlands; 7 Department of Neurology, Máxima Medisch Centrum, Veldhoven, the Netherlands; 8 Department of Neurology, Franciscus Gasthuis & Vlietland, Rotterdam, the Netherlands; 9 Department of Neurology, Elisabeth-TweeSteden Hospital, Tilburg, the Netherlands; 10 Department of Neurology, Haga Hospital, The Hague, the Netherlands; 11 Department of Neurology, St. Antonius Ziekenhuis, Nieuwegein, the Netherlands; 12 Department of Neurology, Amphia Hospital, Breda, the Netherlands; 13 Department of Neurology, Jeroen Bosch Hospital, ‘s-Hertogenbosch, the Netherlands; 14 Department of Neurology, Maastricht University Medical Centre, Cardiovascular Research Institute Maastricht (CARIM), Maastricht, the Netherlands; 15 Department of Neurology, Leiden University Medical Centre, Leiden, the Netherlands; 16 Department of Neurology, University Medical Centre Groningen, Groningen, the Netherlands; 17 Department of Neurology, Amsterdam University Medical Centre, Amsterdam, the Netherlands; 18 Department of Neurology, Albert Schweitzer Hospital, Dordrecht, the Netherlands; 19 Department of Neurology, Zuyderland Hospital, Sittard-Geleen, the Netherlands; 20 Department of Neurology, Frisius Medical Centre, Leeuwarden, the Netherlands; 21 Department of Neurology, Haaglanden Medical Centre, The Hague, the Netherlands; 22 Department of Cardiology, Radboud University Medical Centre, Nijmegen, the Netherlands; 23 Department of Neurology, Erasmus University Medical Centre, Rotterdam, the Netherlands

## Abstract

**Importance:**

Antithrombotic therapy is routinely prescribed to young adults after ischemic stroke to prevent recurrent vascular events. However, the long-term balance between potential benefits and bleeding risks of continued antithrombotic therapy in this population remains uncertain.

**Objective:**

To determine the short-term and long-term risk of bleeding associated with antithrombotic therapy in young stroke survivors.

**Design, Setting, and Participants:**

This cohort study used data from the Observational Dutch Young Symptomatic Stroke Study (ODYSSEY), a prospective, multicenter cohort study conducted in 17 hospitals in the Netherlands. Data were collected between 2013 and 2021. Follow-up data were obtained through standardized questionnaires with subsequent verification based on medical records, with data collection continuing until March 2024. Data were analyzed from October to December 2025. Participants included patients aged 18 to 49 years with a first-ever neuroimaging-proven ischemic stroke who survived the first 30 days after the index event.

**Exposure:**

Antithrombotic therapy.

**Main Outcomes and Measures:**

The primary outcome was the occurrence of a bleeding event requiring medical evaluation. Bleeding complications were stratified by severity according to the Bleeding Academic Research Consortium criteria. Potential factors associated with bleeding risk included sex, age, stroke severity, stroke cause, vascular risk factors, and antithrombotic therapy during follow-up. As a secondary analysis, bleeding risk was compared with the recurrence risk. The estimated bleeding probability during follow-up was calculated using Kaplan-Meier survival analysis with 95% CIs.

**Results:**

In total, 1226 patients were included (median [IQR] age, 44 [38-48] years; 637 male [52.0%]), with a median (IQR) follow-up of 4.2 (2.2-5.9) years. During follow-up, 129 patients experienced at least 1 bleeding event, corresponding to a 5-year cumulative risk of 10.7%. The annual bleeding rate was 2.5 events per 100 person-years. Among patients receiving antiplatelet therapy, the cumulative risk of bleeding at 6 years exceeded that of recurrent vascular events (85 bleeding events [14.7%] vs 105 vascular events [12.9%]). Among patients treated with oral anticoagulants, the cumulative risk of bleeding exceeded the risk of recurrent vascular events at 5 years (22 bleeding events [24.0%] vs 19 vascular events [23.6%]).

**Conclusion and Relevance:**

In this cohort study, young stroke survivors receiving antithrombotic therapy were at substantial long-term risk of bleeding. Although bleeding and recurrent ischemic events may differ in their clinical consequences, these findings highlight that bleeding risk should be carefully considered throughout long-term antithrombotic therapy.

## Key Points

**Question:**

What is the risk of bleeding in young adults (aged 18-49 years) receiving antithrombotic therapy after ischemic stroke?

**Findings:**

In this cohort study of 1226 young patients with ischemic stroke, the 5-year cumulative risk of bleeding was 10.7%, corresponding to an annual bleeding rate of 2.5 events per 100 person-years. Female sex, cardioembolic stroke, and anticoagulant therapy were associated with a higher bleeding risk.

**Meaning:**

These findings indicate that young stroke survivors receiving antiplatelet or anticoagulant therapy face a substantial long-term risk of bleeding, which should be taken into consideration when evaluating the risks and benefits of long-term antithrombotic therapy.

## Teaser

This cohort study examines both short-term and long-term risks of bleeding associated with antithrombotic therapy in young stroke survivors.

## Introduction

An estimated 10% to 15% of all first-ever strokes occur in adults aged 18 to 49 years.^1^ Over the past decades, the number of young adults (18-49 years) affected by ischemic stroke has increased substantially worldwide, in contrast to the declining incidence among individuals older than 50 years.^2^ The growing number of young stroke survivors living with long-term consequences highlights the need to optimize long-term secondary prevention strategies. Antithrombotic treatment, with either antiplatelet agents or anticoagulants, is the cornerstone of secondary prevention after ischemic stroke.^3^ However, long-term use of these agents carries a risk of bleeding.^4,5,6^ This is particularly relevant for young adults, given their prolonged exposure to antithrombotic treatment, as clinical guidelines recommend lifelong continuation of secondary prevention. However, these secondary prevention strategies are based on trials in older populations,^7^ in whom the balance between risks and benefits is better established, while younger patients were underrepresented in these trials.

A previous prospective cohort study^8^ of young patients with ischemic stroke or transient ischemic attack (TIA) showed a 10-year cumulative risk of bleeding requiring evaluation in a hospital setting of 21.8%, which was almost as high as the risk of recurrent ischemic events (33.1%). However, as the study comprised a cohort from 1995 to 2014 and bleeding assessment was retrospective, contemporary prospective data are needed.

Insight in the occurrence of bleeding complications in young adults after ischemic stroke can help to weigh benefits and risks of long-term treatment. In this study, we aimed to determine cumulative bleeding risk and its associated risk factors in a Dutch multicenter cohort of young patients with a first-ever ischemic stroke, and to compare this with the risk of recurrent vascular events.

## Methods

### Study Design and Population

This study is part of the Observational Dutch Young Symptomatic Stroke Study (ODYSSEY), a multicenter, prospective cohort study across 17 centers in the Netherlands, investigating risk factors and prognosis in patients aged 18 to 49 years with a first-ever TIA, ischemic stroke, or intracerebral hemorrhage. The ODYSSEY study was approved by the Medical Review Ethics Committee region Arnhem-Nijmegen, and all patients provided written informed consent. The study was conducted in accordance with the Strengthening the Reporting of Observational Studies in Epidemiology (STROBE) reporting guidelines for cohort studies.

For the present analysis, we included all eligible patients with an index ischemic stroke or TIA between May 2013 and July 2021 who survived the first 30 days after the index event. All patients, including those with transient symptoms (<24 hours), had radiological evidence of cerebral ischemia on computed tomography, magnetic resonance imaging, or vascular imaging (computed tomography angiography or magnetic resonance angiography) demonstrating arterial occlusion. TIAs were, therefore, classified as minor strokes according to the tissue-based definition.^9^ Detailed information regarding data collection procedures have been published previously.^10^

### Baseline Characteristics

Baseline characteristics were obtained at study enrollment and included demographics, vascular risk factors, stroke subtype (TIA or ischemic stroke) and severity, stroke cause, and antithrombotic medication prescribed at discharge or during outpatient evaluation. Stroke severity at admission was assessed with the National Institutes of Health Stroke Scale.^11^ Scores of 3 or less were categorized as minor strokes, and scores greater than 3 as major strokes, according to European Stroke Organisation guidelines.^12,13^ Stroke cause was determined according to the modified Trial of Org 10172 in Acute Stroke Treatment classification,^14^ with the additional subcategory of cervical artery dissection. Antithrombotic therapy was classified by type of agent. Antiplatelet therapy included acetylsalicylic acid, carbasalate calcium, ticagrelor, and clopidogrel; combinations of these agents with dipyridamole were considered a single antiplatelet regimen. Oral anticoagulant therapy comprised vitamin K antagonists and direct oral anticoagulants. Other antithrombotic therapy included low-molecular-weight heparin and combinations of antithrombotic agents, whereas combinations of antiplatelet agents were classified as dual antiplatelet therapy (DAPT).

### Definition of Risk Factors

We included the following risk factors in our analyses: hypertension, diabetes, dyslipidemia, smoking, and alcohol use. Hypertension was defined as a known history of hypertension, use of antihypertensive medication, or elevated blood pressure measured at least 24 hours after the index event, with a systolic blood pressure greater than 140 mm Hg and/or a diastolic blood pressure greater than 90 mm Hg. Diabetes was defined as a known history of diabetes, use of antidiabetic medication, 2 separate fasting glucose measurements greater than 126.1 mg/dL (to convert to millimoles per liter, multiply by 0.0555), or a single hemoglobin A_1c_ measurement greater than 6.5% (to convert to proportion of total hemoglobin, multiply by 0.01). Dyslipidemia was defined according to the modified Trial of Org 10172 in Acute Stroke Treatment classification, encompassing a known history of dyslipidemia, use of statins, or elevated low-density lipoprotein cholesterol levels greater than 158.3 mg/dL (to convert to millimoles per liter, multiply by 0.0259). Smoking status was categorized as current, previous, or never. Alcohol use was assessed according to self-reported consumption, with alcohol abuse defined as more than 20 units per week (ie, >200 g of alcohol per week, according to the modified International Prognostic Scoring System criteria).^14^

### Antithrombotic Therapy During Follow-Up

Antithrombotic therapy was also assessed during follow-up to account for treatment changes over time. For patients who switched regimens, the date of change was recorded. If the exact date of therapy change was unknown, it was approximated as the midpoint between the index event and the first follow-up, or between 2 follow-up visits during which the therapy change was reported. For patients who changed from DAPT to antiplatelet monotherapy, a consecutive sample from the coordinating center showed a median duration of DAPT of 21 days, consistent with current stroke guideline recommendations.^13^ We, therefore, applied this duration of DAPT to all other patients switching from DAPT to antiplatelet monotherapy when the exact date of therapy change was unknown.

### Outcome Assessment

The primary outcome was defined as a bleeding event requiring medical evaluation, including emergency department visits, hospitalization, outpatient consultation, or evaluation by the general practitioner (GP) that resulted in any form of treatment to manage bleeding. Bleeding events were primarily collected through structured annual follow-up questionnaires via telephone or email. When a bleeding event was reported, the corresponding hospital medical records were reviewed. If no matching event was identified, the patient’s GP was contacted for information to verify the event. Data collection continued until March 2024. Only events that were evaluated and documented by a medical specialist or GP were included. Each bleeding event was classified according to the Bleeding Academic Research Consortium (BARC) criteria,^15^ which range from no bleeding (type 0) to fatal bleeding (type 5). We defined minor bleeding as BARC 2-3a, and major bleeding as BARC 3b-5 (eTable 1 in Supplement 1).^8^ Major bleeding is distinguished from minor bleeding mainly by the requirement for surgical intervention to control bleeding. Additionally, bleeding events were categorized by type: spontaneous, trauma related, or secondary to surgery (included only if unexpected for the specific surgical procedure). Moreover, bleeds were classified according to anatomical location: intracranial, intraocular, nasal, gastrointestinal, pulmonary, urogenital, gynecologic, and soft tissue. All bleeding events were assessed and rated by clinician-scientists (A.R., E.R., F.E.d.L., and N.A.H.) from the Radboud University Medical Center, and a consensus meeting was held to ensure consistency in scoring. Antithrombotic therapy at the time of the bleeding event was recorded when available; otherwise, the regimen prescribed at the most recent follow-up prior to the event was used for analysis.

In addition, we assessed the cumulative risk of recurrent vascular events per antithrombotic subgroup, defined as recurrent ischemic stroke, TIA, myocardial infarction, revascularization procedures, or vascular death, as recorded in the ODYSSEY study between November 2013 and June 2022. Vascular death was defined as death within 30 days of a recurrent vascular event. The analysis of recurrent vascular events in the ODYSSEY population has been reported previously.^16^

### Statistical Analysis

We calculated the estimated bleeding probability during follow-up using Kaplan-Meier survival analysis with 95% CIs. Patients were censored at the end of follow-up or last available contact. The annual rate of bleeding was calculated as the number of events per 100 person-years, accounting for the population at risk in each year. We stratified analyses by sex, age category, stroke subtype and severity, stroke cause, and type of antithrombotic therapy at discharge and during follow-up. Antithrombotic therapy during follow-up was analyzed as a time-dependent variable.

To assess differences in cumulative bleeding risk across subgroups, pairwise log-rank tests were performed. For variables with more than 2 categories, the Holm-Bonferroni method was used to account for multiple comparisons.

We performed univariable and multivariable Cox proportional hazards regression to identify factors associated with bleeding. We included variables with *P* < .20 from univariable analyses in the multivariable model and verified the proportional hazards assumption using Schoenfeld residuals. Analyses were based on complete cases. Sensitivity analyses were performed by excluding gynecologic bleeding events and restricting the outcome to major bleeding events (BARC 3b-5). We conducted all statistical analyses from October to December 2025 using R software version 2024.04 (R Project for Statistical Computing) and considered 2-sided *P* < .05 statistically significant unless the Bonferroni-Holm correction was applicable.

## Results

### Patient Characteristics

A total of 1226 patients were included in the study, with a median (IQR) age of 44 (38-48) years at the time of the index event (eFigure in Supplement 1). The cohort comprised 589 women (48.0%) and 637 men (52.0%). Patient characteristics are summarized in Table 1. The median (IQR) follow-up duration was 4.2 (2.2-5.9) years, with a total of 5113 person-years of follow-up. Baseline characteristics according to follow-up status are provided in eTable 2 in Supplement 1.

**Table 1.** Patient Characteristics at Baseline

Characteristic	Total No. (N = 1226)	Patients, No. (%)		*P* value
		Without bleeding complication (n = 1097)	With bleeding complication (n = 129)	
Patient characteristics				
Diagnosis				
Transient ischemic attack	95	87 (91.6)	8 (8.4)	.60
Ischemic stroke	1131	1010 (89.3)	121 (10.7)	
Age, median (IQR), y	44 (38-47)	44 (38-47)	45 (40-48)	.25
Age group, y				
18-29	126	113 (89.7)	13 (10.3)	.34
30-34	92	86 (93.5)	6 (6.5)	
35-39	155	143 (92.3)	12 (7.7)	
40-44	297	259 (87.2)	38 (12.8)	
45-49	556	496 (89.2)	60 (10.8)	
Sex				
Male	637	601 (94.3)	36 (5.7)	<.001
Female	589	496 (84.2)	93 (15.8)	
Stroke characteristics				
Trial of Org 10172 in Acute Stroke Treatment stroke classification				
Atherothrombotic	52	48 (92.3)	4 (7.7)	.03
Likely atherothrombotic	171	153 (89.5)	18 (10.5)	
Small vessel disease	167	157 (94.0)	10 (6.0)	
Cardioembolic^a^	198	165 (83.3)	33 (16.7)	
Antiplatelet therapy	156	132 (84.6)	24 (15.4)	
Oral anticoagulants or other therapy	40	31 (77.5)	9 (22.5)	
Rare cause	130	116 (89.2)	14 (10.8)	
Cervical artery dissection	144	134 (93.1)	10 (6.9)	
Cryptogenic	307	276 (89.9)	31 (10.1)	
Multiple causes	57	48 (84.2)	9 (15.8)	
National Institutes of Health Stroke Scale score at admission, median (IQR)^b^	3 (1-6)	3 (1-6)	3 (1-7)	.39
≤3 (Minor stroke)	732	658 (89.9)	74 (10.1)	.60
>3 (Major stroke)	490	435 (88.8)	55 (11.2)	
Vascular risk factors				
Hypertension^c^	464	410 (88.4)	54 (11.6)	.37
Diabetes^d^	117	108 (92.3)	9 (7.7)	.37
Dyslipidemia^e^	249	225 (90.4)	24 (9.6)	.69
Alcohol abuse	76	69 (90.8)	7 (9.2)	.85
Smoking status				
Current	273	241 (88.3)	32 (11.7)	.24
Never	385	337 (87.5)	48 (12.5)	
Previous	345	316 (91.6)	29 (8.4)	
Unknown	223	203 (91.0)	20 (9.0)	
Secondary prevention: antithrombotic therapy at discharge^f^				
Antiplatelet	955	849 (88.9)	106 (11.1)	.006
Dual antiplatelet	166	158 (95.2)	8 (4.8)	
Anticoagulant	62	49 (79.0)	13 (21.0)	
Other	29	28 (96.6)	1 (3.4)	
None	8	7 (87.5)	1 (12.5)	

^a^ Two patients with cardioembolic stroke cause did not receive any antithrombotic therapy at discharge.

^b^ National Institutes of Health Stroke Scale score at admission was not available for 4 patients.

^c^ Defined as known history of hypertension, use of antihypertensive medication, a systolic blood pressure greater than 140 mm Hg, and/or a diastolic blood pressure greater than 90 mm Hg at least 24 hours after index event.

^d^ Defined as known history of diabetes, use of diabetic medication, 2 values of glucose greater than 126.1 mg/dL (to convert to millimoles per liter, multiply by 0.0555) or 1 hemoglobin A_1c_ measurement greater than 6.5% (to convert to proportion of total hemoglobin, multiply by 0.01).

^e^ Defined as known history of dyslipidemia, use of statins, or low-density lipoprotein cholesterol greater than 158.3 mg/dL (to convert to millimoles per liter, multiply by 0.0259).

^f^ Information about antithrombotic therapy at discharge was not available for 6 patients.

Among patients with available data, at discharge, 1121 (91.9%) were treated with antiplatelet therapy, of whom 166 (13.6%) received DAPT. Oral anticoagulant therapy was prescribed at discharge in 5.1% of cases (62 patients), whereas 2.4% (29 patients) received antithrombotic treatment in the form of low-molecular-weight heparin or combination regimens. For 8 patients, no antithrombotic therapy was started. These mainly involved nonatherosclerotic stroke causes, including hematological malignant neoplasm (1 patient), benign hematological disease (2 patients), cirrhosis-related thrombocytopenia (1 patient), reversible cerebral vasoconstriction syndrome (1 patient), and posttraumatic fat embolism (1 patient). One case was initially classified as a stroke mimic, and another had an unknown reason for withholding therapy. eTables 3 and 4 in Supplement 1 provide an overview of antithrombotic therapy during follow-up.

### Incidence of Bleeding Events

**Figure 1.**
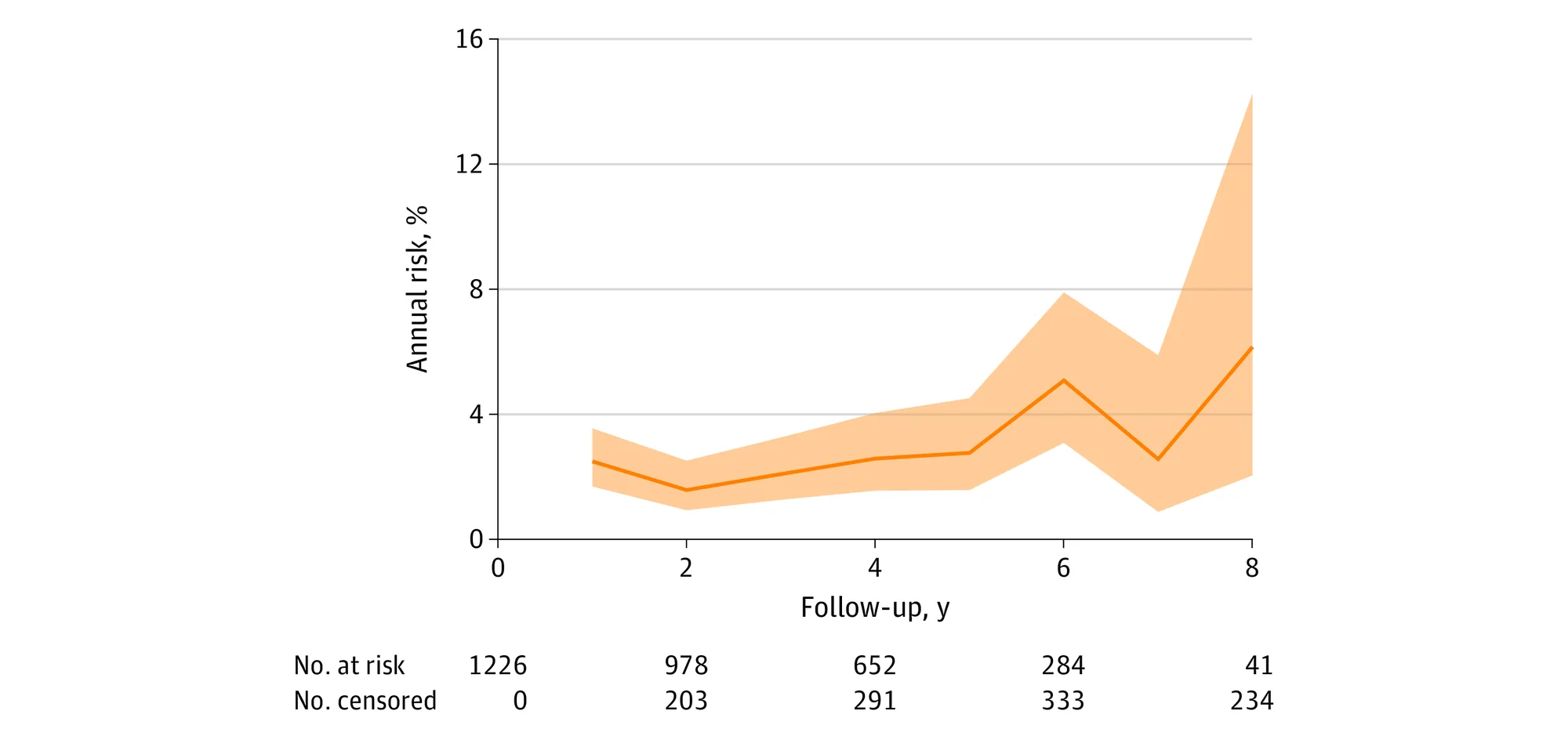
Line Graph of Annual Risk of Bleeding The incidence rate per 100 person-years was calculated by dividing the number of events by the total person-years at risk per 1-year interval. Shaded areas indicate 95% CI.

A total of 145 bleeding events occurred in 129 patients. Of these, 15 patients (11.6%) experienced more than 1 bleeding event. Detailed information on the type, location, and severity of bleeding events is provided in Table 2. The overall incidence rate of bleeding was 2.5 events per 100 patient-years. Annual risks are presented in Figure 1. The estimated 5-year probability of bleeding was 10.7%, corresponding to an annual risk of 2.0% to 2.5%. The overall bleeding rate was 2.3 events per 100 person-years among patients receiving antiplatelet therapy, compared with 5.2 events per 100 person-years among those receiving oral anticoagulants. The incidence rate of bleeding was highest during the first 30 days after the index event (3.0 events per 100 person-years; 95% CI, 0.62-8.72 events per 100 person-years), compared with 2.6 events per 100 person-years between 30 days and 6 months (95% CI, 1.51-4.29 events per 100 person-years), and 2.4 events per 100 person-years between 6 months and 1 year (95% CI, 1.62-3.48 events per 100 person-years).

**Table 2.** Bleeding Events During Follow-Up

Characteristic	Total No. of events	Patients, No. (%)	
		Male	Female
Bleeding events	145	39 (26.9)	106 (73.1)
Occurrence			
Spontaneous	135	35 (25.9)	100 (74.1)
Traumatic	7	2 (28.6)	5 (71.4)
Surgery related	3	2 (66.7)	1 (33.3)
Localization			
Gynecologic	75	0	75 (100.0)
Nasal	11	6 (54.5)	5 (45.5)
Gastrointestinal	34	19 (55.9)	15 (44.1)
Urological	9	6 (66.7)	3 (33.3)
Pulmonal	1	0	1 (100.0)
Soft tissue	7	2 (28.6)	5 (71.4)
Intraocular	1	1 (100.0)	0
Intracranial	7	5 (71.4)	2 (28.6)
Severity^a^			
Minor			
Type 2	110	30 (27.3)	80 (72.7)
Type 3a	26	4 (15.4)	22 (84.6)
Major			
Type 3b	2	0	2 (100.0)
Type 3c	7	5 (71.4)	2 (28.6)

^a^ Denotes severity according to Bleeding Academic Research Consortium classification.

**Figure 2.**
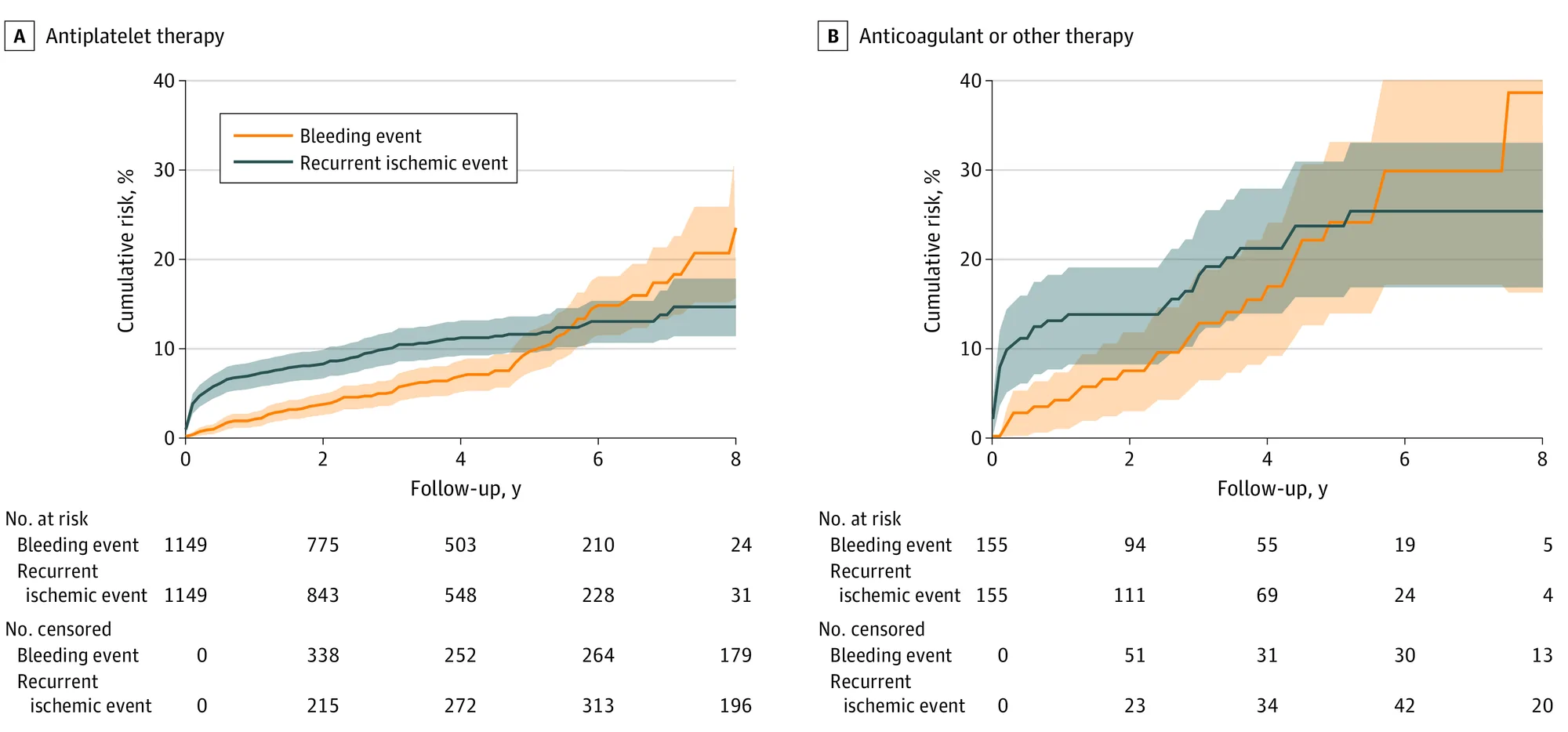
Kaplan-Meier Graphs of Cumulative Risk of Bleeding and Recurrent Ischemic Events Panel A shows data for patients using antiplatelet therapy. Panel B shows data for patients using anticoagulant or other therapy (ie, low-molecular-weight heparin or combinations of antithrombotic treatment). Shaded areas indicate 95% CIs.

Cumulative risks of bleeding and recurrent vascular events, stratified by antithrombotic treatment regimen—antiplatelet therapy (single or dual) and oral anticoagulant/other therapy—are shown in Figure 2. At 6 years, patients receiving antiplatelet therapy had a higher cumulative bleeding risk than the risk of recurrent vascular events (85 bleeding events [14.7%] vs 105 vascular events [12.9%]) (Figure 2A). Among patients treated with oral anticoagulants, a similar trend was observed, with bleeding risk exceeding vascular recurrence risk at 5 years (22 bleeding events [24.0%] vs 19 vascular events [23.6%]) (Figure 2B).

Women had a higher risk for bleeding complications compared with men, primarily due to the high incidence of gynecologic bleeding (3.1 events per 100 person-years). Management of gynecologic bleeding included intrauterine device placement (15 patients), endometrial ablation (10 patients), hormonal therapy (6 patients), and iron supplementation or transfusion (8 patients); for 26 patients, no intervention was performed or documented.

Among patients with cryptogenic stroke cause, the bleeding risk was 3.4 events per 100 person-years in the first year and 2.6 events per 100 person-years beyond 3 years. Nine patients (6.2%) experienced a major bleed, including 7 cases of intracranial hemorrhage, 2 of which were surgery related (epidural hematoma after bone flap replacement and intracranial hemorrhage after spine surgery, 1 patient each), and 2 gynecologic bleeds, both requiring surgical intervention. No deaths were due to bleeding events, whereas 2 patients died from recurrent vascular events.

### Variables Associated With Bleeding

In univariable analysis, sex, stroke cause, smoking, and antithrombotic therapy during follow-up were associated with increased bleeding risk (Table 3). Multivariable analysis showed that female sex (hazard ratio [HR], 3.39; 95% CI, 2.12-5.41; *P* < .001), cardioembolic stroke cause (HR, 2.17; 95% CI, 1.14-4.14; *P* = .02), and anticoagulant therapy (HR, 3.07; 95% CI, 1.34-7.07; *P* = .008) were independently associated with an increased risk of bleeding.

**Table 3.** Univariable and Multivariable Cox Proportional Hazards Regression

Factor	Univariable analysis		Multivariable analysis	
	HR (95% CI)	*P* value	HR (95% CI)	*P* value
Event type				
Transient ischemic attack	1 [Reference]	NA	1 [Reference]	NA
Ischemic stroke	1.36 (0.66-2.80)	.41	NA	NA
Age group, y				
18-29	1 [Reference]	NA	1 [Reference]	NA
30-34	0.59 (0.22-1.56)	.29	NA	NA
35-39	0.72 (0.31-1.67)	.44	NA	NA
40-44	1.17 (0.61-2.25)	.64	NA	NA
45-49	0.97 (0.52-1.81)	.92	NA	NA
Sex				
Male	1 [Reference]	NA	1 [Reference]	NA
Female	3.09 (2.07-4.63)	<.001	3.39 (2.12-5.41)	<.001
TOAST stroke classification				
Atherothrombotic	0.70 (0.25-1.97)	.49	1.26 (0.44-3.65)	.67
Likely atherothrombotic	1.09 (0.60-1.99)	.77	1.45 (0.74-2.83)	.28
Small vessel disease	0.61 (0.29-1.28)	.20	0.99 (0.46-2.14)	.98
Cardioembolic	2.25 (1.34-3.80)	.002	2.17 (1.14-4.14)	.02
Rare cause	1.22 (0.63-2.38)	.55	1.12 (0.54-2.31)	.75
Cervical artery dissection	0.85 (0.41-1.79)	.68	1.29 (0.55-3.00)	.56
Cryptogenic	1 [Reference]	NA	1 [Reference]	NA
Multiple causes	2.27 (1.03-5.02)	.04	2.18 (0.88-5.40)	.09
National Institutes of Health Stroke Scale score at admission				
≤3 (Minor stroke)	1 [Reference]	NA	1 [Reference]	NA
>3 (Major stroke)	1.18 (0.82-1.71)	.38	NA	NA
Vascular risk factors				
Hypertension^a^	1.13 (0.78-1.64)	.50	NA	NA
Diabetes^b^	0.66 (0.33-1.31)	.23	NA	NA
Dyslipidemia (according to TOAST criteria)	0.95 (0.59-1.52)	.83	NA	NA
Alcohol abuse	0.72 (0.32-1.61)	.42	NA	NA
Smoking status				
Ever	0.75 (0.50-1.11)	.15	0.71 (0.48-1.07)	.10
Never	1 [Reference]	NA	1 [Reference]	NA
Antithrombotic therapy at follow-up				
None	1 [Reference]	NA	1 [Reference]	NA
Antiplatelet	1.77 (0.89-3.53)	.10	1.95 (0.89-4.28)	.10
Anticoagulant	3.77 (1.74-8.19)	<.001	3.07 (1.34-7.07)	.008
Other	3.22 (0.88-11.81)	.08	3.16 (0.90-11.07)	.07

Abbreviations: HR, hazard ratio; NA, not applicable; TOAST, Trial of Org 10172 in Acute Stroke Treatment.

^a^ Defined as known history of hypertension, use of antihypertensive medication, a systolic blood pressure greater than 140 mm Hg, and/or a diastolic blood pressure greater than 90 mm Hg at least 24 hours after index event.

^b^ Defined as known history of diabetes, use of diabetic medication, 2 values of glucose greater than 126.1 mg/dL (to convert to millimoles per liter, multiply by 0.0555), or 1 hemoglobin A_1c_ measurement greater than 6.5% (to convert to proportion of total hemoglobin, multiply by 0.01).

In sensitivity analyses excluding gynecologic bleeding events (54 bleeding events remaining), female sex was not significantly associated with bleeding (HR, 1.35; 95% CI, 0.77-2.37; *P* = .30). Similarly, when restricting the outcome to major bleeding events (9 events remaining), no association between female sex and bleeding was observed (HR, 0.90; 95% CI, 0.23-3.58; *P* = .89).

Only 21% of patients with cardioembolic stroke (42 patients) received oral anticoagulants during follow-up, as most had a patent foramen ovale as the presumed source and were treated with antiplatelet therapy. In a subgroup analysis restricted to patients receiving antiplatelet therapy throughout follow-up, cardioembolic cause remained associated with increased bleeding risk (HR, 2.27; 95% CI 1.20-4.30; *P* = .01).

## Discussion

In this cohort study, we found a 5-year cumulative risk of bleeding of 10.7%, corresponding to an annual risk of 2.0% to 2.5%, with no decline over time. Women had a higher incidence of bleeding compared with men, primarily driven by frequent gynecologic bleeds. Both anticoagulant therapy and cardioembolic stroke cause were independently associated with increased bleeding risk, with the latter not readily explained by other more prevalent risk factors in this subgroup. Major bleeding events were infrequent, and no deaths were directly attributable to bleeding. None of the patients discontinued antithrombotic therapy directly due to bleeding.

Our findings align with prior research on bleeding risk in young patients (aged 18-49 years) with ischemic stroke. A previous Dutch cohort study^8^ reported a cumulative bleeding risk of 21.8% over 10 years, with female individuals at higher risk.

In older populations (aged >50 years), the safety of antithrombotic therapy has also been studied. In a combined analysis of 6 trials^17^ evaluating adverse events of long-term antiplatelet therapy (mean age, 65.5 years; 82 199 person-years of follow-up), the risk of major bleeding was highest within 30 days after the index event (2.5 events per 100 person-years for clopidogrel and 5.8 events per 100 person-years for DAPT), consistent with our findings.

Our results suggest that the bleeding risk associated with oral anticoagulants is similar in younger and older patients. The overall bleeding rate among patients receiving oral anticoagulants was 5.2 events per 100 person-years, comparable to the rate of 5.3 per 100 person-years reported in a study^18^ of patients with a mean age of 72.2 years treated with direct oral anticoagulants for heart failure or atrial fibrillation. This comparable risk may be explained by the high incidence of gynecologic bleeding in our cohort, which substantially contributed to the overall bleeding burden, as well as the inclusion of all bleeding events requiring medical attention rather than only major bleeding events.

None of these studies in older populations reported significant sex differences in bleeding occurrence. This discrepancy may be explained by the high frequency of gynecologic bleeding events in women in our cohort, which are more common during the reproductive years. Heavy menstrual bleeding can substantially impact quality of life, as demonstrated in a study^19^ of 1547 Swedish women aged 40 to 45 years, among whom those with heavy menstrual bleeding reported worse quality of life across all domains of the 36-item Short Form Health Survey questionnaire.

Compared with the general population, risk of gynecologic bleeding is increased among premenopausal women taking antithrombotic therapy. A Dutch retrospective cohort study^20^ of women of reproductive age, based on a general practice consultation registry, reported an average annual incidence of heavy menstrual bleeding requiring GP consultation of 0.9 events per 100 person-years. In comparison, our cohort showed an estimated incidence of gynecologic bleeding events of 3.1 events per 100 person-years among women receiving antithrombotic therapy.

According to current guidelines, antiplatelet therapy is recommended for patients with noncardioembolic stroke. A meta-analysis^7^ of 18 720 patients with a history of ischemic stroke or TIA from 21 trials, allocated to antiplatelet therapy for a mean duration of 29 months, demonstrated an absolute risk reduction of 36 events per 1000 patients in preventing recurrent vascular events, corresponding to a number needed to treat of approximately 28. The benefit of antiplatelet therapy has been shown to outweigh the risk of bleeding associated with antiplatelet therapy in older patients, although the included trials generally had follow-up durations of only 2 to 3 years. In younger populations, the balance between lifelong benefit and bleeding risk remains uncertain, particularly for patients with cryptogenic stroke, for whom recurrence risk declines over time (0.5% per year beyond 3 years).^16^ In contrast, bleeding risk persists, averaging 2.6 events per 100 person-years after 3 years of antiplatelet therapy.^16^ This emphasizes the importance of carefully evaluating the risks of long-term antiplatelet therapy, as the benefit-risk balance may shift over time.^16^

Although a recurrent ischemic stroke is often perceived as a more severe, disabling event than a bleeding complication, functional stroke recovery in young patients is generally favorable, with most achieving good long-term neurological outcomes assessed by the modified Rankin Scale score.^14,21^ Nevertheless, long-term psychosocial symptoms after ischemic stroke at a young age are common.^22,23,24^ However, bleeding complications can substantially impact quality of life as well.^19,25^ Considering both ischemic and bleeding risks from the patient’s perspective is crucial, as the experience of bleeding may affect adherence to therapy. In patients after myocardial infarction, preferences regarding antithrombotic treatment were strongly influenced by bleeding risk: patients were willing to accept considerable increases in cardiovascular mortality risk to avoid nonfatal bleeding events.^26^ This highlights the need to quantify bleeding risks for personalized counseling and shared decision-making in long-term antithrombotic therapy.

Given the uncertainty regarding the optimal duration of antiplatelet therapy in young patients with cryptogenic stroke, we designed the STOP trial.^27^ This trial evaluates whether discontinuation of antiplatelet therapy is noninferior to continuation for preventing recurrent vascular events, with major bleeding as a secondary outcome. In addition, the HALTI trial^28^ investigates whether antiplatelet therapy can safely be stopped 12 months after patent foramen ovale closure in young patients with cryptogenic stroke. Both studies are currently recruiting.

### Limitations

Several limitations of this study should be acknowledged. Bleeding and ischemic complications may have been underreported if patients failed to report events in follow-up questionnaires, potentially resulting in information and recall bias and underestimation of risks. In addition, data on medication adherence, severity of recurrent vascular events, and functional outcomes after recurrent stroke and bleeding events were not available.

## Conclusions

In this multicenter cohort study, young patients with ischemic stroke had a substantial risk of bleeding during long-term antithrombotic therapy. Female sex, anticoagulant therapy, and cardioembolic stroke were key variables. These findings highlight the importance of recognizing bleeding risks associated with long-term antithrombotic therapy.

## Notes

Supplement 1. eFigure. Study population eTable 1. Bleeding Academic Research Consortium (BARC) criteria eTable 2. Baseline characteristics of participants with and without follow-up data eTable 3. Use of antithrombotic therapy in years 1 and 3 of follow-up eTable 4. Antithrombotic therapy during follow-up expressed in person-years and % of follow-up

Supplement 2. Data Sharing Statement
