# Supplementary material for: Antithrombotic Therapy and Bleeding Risk Among Young Adults After Ischemic Stroke
Source: JAMA Netw Open. 2026 Sep 24;9(9):e2635826. doi: 10.1001/jamanetworkopen.2026.35826 (PMC13613175; doi:10.1001/jamanetworkopen.2026.35826)
Supplement: Supplementary file 1 [file jamanetwopen-e2635826-s001.pdf]

Supplemental Online Content

Rasing A, Verburgt E, Ekker MS, et al. Antithrombotic therapy and bleeding risk among young adults after ischemic stroke. *JAMA Netw Open*. 2026;9(9):e2635826.
doi:10.1001/jamanetworkopen.2026.35826

eFigure. Study population

eTable 1. Bleeding Academic Research Consortium (BARC) criteria

eTable 2. Baseline characteristics of participants with and without follow-up data

eTable 3. Use of antithrombotic therapy in years 1 and 3 of follow-up

eTable 4. Antithrombotic therapy during follow-up expressed in person-years and % of follow-up

This supplemental material has been provided by the authors to give readers additional information about their work.

© 2026 Rasing A et al. *JAMA Network Open*.

eFigure. Study population

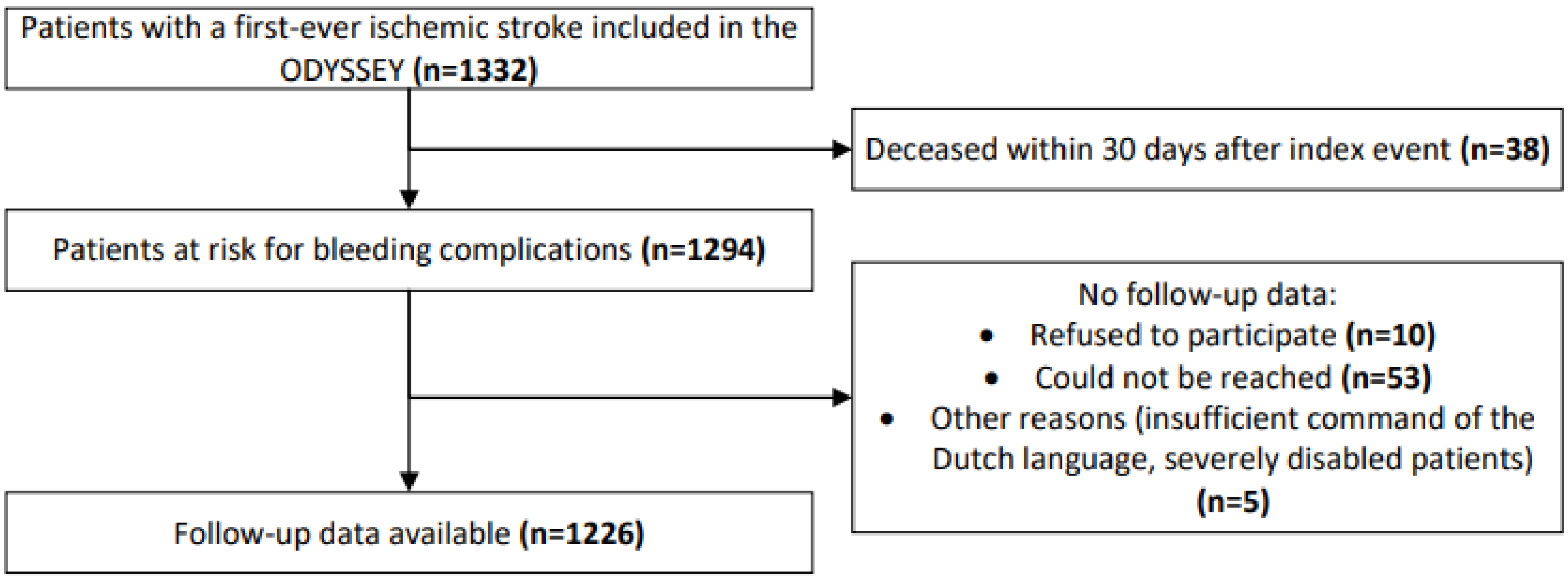

© 2026 Rasing A et al. *JAMA Network Open*.

eTable 1. Bleeding Academic Research Consortium (BARC) criteria.

BARC type	Severity	Definition
Type 0	NA	No bleeding
Type 1	NA	Bleeding that is not actionable and does not cause the patient to seek unscheduled performance of studies, hospitalization, or treatment by a healthcare professional; may include episodes leading to self-discontinuation of medical therapy by the patient without consulting a healthcare professional.
Type 2	Minor	Any overt, actionable sign of hemorrhage (e.g., more bleeding than would be expected for a clinical circumstance, including bleeding found by imaging alone) that does not fit the criteria for type 3, 4, or 5 but does meet at least one of the following criteria: (1) requiring nonsurgical, medical intervention by a healthcare professional, (2) leading to hospitalization or increased level of care, or (3) prompting evaluation.
Type 3a	Minor	Overt bleeding plus hemoglobin drop of 3 to <5 g/dL (provided hemoglobin drop is related to bleed) and/or any transfusion with overt bleeding.
Type 3b	Major	Overt bleeding plus hemoglobin drop ≥ 5 g/dL (provided hemoglobin drop is related to bleed) and/or cardiac tamponade and/or bleeding requiring surgical intervention for control (excluding dental/nasal/skin/hemorrhoid) and/or bleeding requiring intravenous vasoactive agents.
Type 3c	Major	Intracranial hemorrhage (does not include microbleeds or hemorrhagic transformation, does include intraspinal) and/or subcategories confirmed by autopsy or imaging or lumbar puncture and/or intraocular bleed compromising vision.
Type 4	Major	CABG-related bleeding: Perioperative intracranial bleeding within 48 hours and/or reoperation after closure of sternotomy for the purpose of controlling bleeding and/or transfusion of ≥ 5 U whole blood or packed red blood cells within a 48-hour period and/or chest tube output of ≥ 2L within a 24-hour period.
Type 5	Major	Fatal bleeding

© 2026 Rasing A et al. *JAMA Network Open*.

eTable 2. Baseline characteristics of participants with and without follow-up data

Characteristic	With follow-up	Without follow-up
No. Patients	1226	68
**Patient characteristics**		
TIA	95 (7.7)	2 (2.9)
Ischemic stroke	1131 (92.3)	66 (97.1)
Age, median (IQR), y	44 [38-47]	44 [38-47]
18-29 y	126 (10.3)	3 (4.4)
30-34 y	92 (7.5)	10 (14.7)
35-39 y	155 (12.6)	6 (8.8)
40-44 y	297 (24.2)	18 (26.5)
45-49 y	556 (45.4)	31 (45.6)
Sex		
Male	637 (52.0)	40 (58.8)
Female	589 (48.0)	28 (41.2)
**Stroke characteristics**		
TOAST stroke classification		
Atherothrombotic	52 (4.2)	2 (2.9)
Likely atherothrombotic	171 (13.9)	16 (23.5)
Small vessel disease	167 (13.6)	7 (10.3)
Cardioembolic	198 (16.2)	11 (16.2)
Rare cause	130 (10.6)	7 (10.3)
Cervical artery dissection	144 (11.7)	5 (7.4)
Cryptogenic	307 (25.0)	18 (26.5)
Multiple causes	57 (4.6)	2 (2.9)
NIHSS score at admission, median (IQR)[a]	3 [1-6]	3 [2-8]
≤3 (minor stroke)	732 (59.7)	36 (52.9)
>3 (major stroke)	490 (40.0)	32 (47.1)

© 2026 Rasing A et al. *JAMA Network Open*.

**Vascular risk factors**		
Hypertension[b]	464 (37.8)	29 (42.6)
Diabetes[c]	117 (9.5)	8 (11.8)
Dyslipidemia[d]	249 (20.3)	14 (20.6)
Alcohol abuse	76 (6.2)	8 (11.8)
Smoking status		
Current	273 (22.3)	20 (29.4)
Never	385 (31.4)	10 (14.7)
Previous	345 (28.1)	17 (25.0)
Unknown	223 (18.2)	21 (30.9)
**Secondary prevention**		
Antithrombotic therapy at discharge[e]		
Antiplatelet	955 (77.9)	50 (73.5)
Dual antiplatelet	166 (13.5)	13 (19.1)
Anticoagulant	62 (5.1)	3 (4.4)
Other	29 (2.4)	1 (1.5)
None	8 (0.7)	1 (1.5)

Abbreviations: IQR = interquartile range; TIA = transient ischemic attack; NIHSS = National Institutes of Health Stroke Scale; TOAST = Trial of Org 10172 in Acute Stroke Treatment. Data are n (%) or median (IQR). *a* NIHSS at admission was not available for 4 patients; *b* known history of hypertension, use of antihypertensive medication, a systolic blood pressure >140 mm Hg, and/or a diastolic blood pressure >90 mm Hg at least 24 hours after index event; *c* known history of diabetes, use of diabetic medication, 2 values of glucose greater than 126.1 mg/dL (to convert to millimoles per liter, multiply by 0.0555) or 1 hemoglobin A_{1c} measurement greater than 6.5% (to convert to proportion of total hemoglobin, multiply by 0.01); *d* known history of dyslipidemia, use of statins, low-density lipoprotein cholesterol greater than 158.3 mg/dL (to convert to millimoles per liter, multiply by 0.0259); *e* Information about antithrombotic therapy at discharge was not available for 6 patients.

© 2026 Rasing A et al. *JAMA Network Open*.

eTable 3. Use of antithrombotic therapy in years 1 and 3 of follow-up.

Years	None (%)	Antiplatelet (%)	Dual antiplatelet (%)	Oral anticoagulant (%)	Other (%)	Unknown (%)
0 (discharge)	8 (0.7)	955 (77.9)	166 (13.5)	62 (5.1)	29 (2.4)	6 (0.5)
1	72 (6.2)	940 (80.7)	31 (2.7)	105 (9.0)	14 (1.2)	3 (0.3)
3	91 (10.9)	647 (77.6)	14 (1.7)	73 (8.8)	9 (1.1)	0 (0.0)

At 1 year follow-up, 61 patients were censored due to loss to follow-up or a bleeding event. After three years, 392 were censored due to loss to follow-up or a bleeding event.

eTable 4. Antithrombotic therapy during follow-up expressed in person-years and % of follow-up.

Antithrombotic therapy	Person-years of follow-up	% of total follow-up	Number of bleeding event	Event rate per 100 person years
None	484	9.5	8	1.7
Antiplatelet	4007	78.4	93	2.3
Dual antiplatelet	107	2.1	2	1.9
Anticoagulant	444	8.7	23	5.2
Other	64	1.3	3	4.7
Unknown	7	0.1	0	0.0

© 2026 Rasing A et al. *JAMA Network Open*.
