# Supplementary material for: Antithrombotic Therapy and Bleeding Risk Among Young Adults After Ischemic Stroke
Source: JAMA Netw Open. 2026 Sep 24;9(9):e2635826. doi: 10.1001/jamanetworkopen.2026.35826 (PMC13613175; doi:10.1001/jamanetworkopen.2026.35826)
Supplement: Supplementary file 2 [file jamanetwopen-e2635826-s002.pdf]

Data Sharing Statement

Rasing. Antithrombotic Therapy and Bleeding Risk Among Young Adults After Ischemic Stroke. *JAMA Netw Open*. Published September 24, 2026. doi:10.1001/jamanetworkopen.2026.35826

Data
Data available: Yes
Data types: Other (please specify)
Additional Information: Data is available upon request. Requests should be sent to the corresponding author for further information.
How to access data: Data is available upon request. Requests should be sent to the corresponding author for further information.
When available: With publication

Supporting Documents
Document types: Other (please specify)
Additional Information: Data is available upon request. Requests should be sent to the corresponding author for further information.
How to access documents: Data is available upon request. Requests should be sent to the corresponding author for further information.
When available: With publication

Additional Information
Who can access the data: Data is available upon request. Requests should be sent to the corresponding author for further information.
Types of analyses: Data is available upon request. Requests should be sent to the corresponding author for further information.
Mechanisms of data availability: Data is available upon request. Requests should be sent to the corresponding author for further information.
Any additional restrictions: Data is available upon request. Requests should be sent to the corresponding author for further information.
